# Swiss flag or Red Cross emblem: why the confusion?

**DOI:** 10.1186/1754-9493-7-13

**Published:** 2013-05-07

**Authors:** Philip F Stahel

**Affiliations:** 1 Department of Orthopaedics, and Department of Neurosurgery, University of Colorado, School of Medicine, Denver Health Medical Center, 777 Bannock Street, Denver, CO 80204, USA; 2 Department of Neurosurgery, University of Colorado, School of Medicine, Denver Health Medical Center, 777 Bannock Street, Denver, CO 80204, USA

## 

As a dual Swiss-US citizen, I have been astonished for many years about the sheer quantity of Swiss National flags decorating the United States. Indeed, I encounter the Swiss flag regularly in football stadiums, ballparks, and concert arenas, owing to the unequivocal earmarking of first aid stations and first aid responders at popular venues (Figure 1). The root cause is likely attributed to an unintentional mix-up of the Swiss flag with the Red Cross emblem. This misconception appears to be increasingly prevalent throughout the United States. In general, I would deem such a trivial oversight as unworthy of a formal correction. However, I was recently stunned to notice that the new Emergency Room of our own University Hospital in Colorado [1] is also “misbranded” with the Swiss cross (Figure 2). This most recent anecdotal episode set the incentive for the drafting of this editorial. Clearly, the purpose of this article is not meant to be condescending or indoctrinating, but rather aimed at shedding some light on the interesting historic background on the origin of the Swiss flag and Red Cross, and to potentially clarify a widespread misperception.

**Figure 1:**
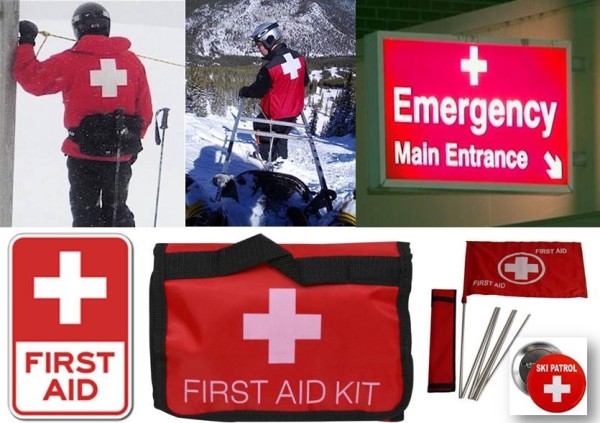
Erroneous “misbranding” of medical services with the Swiss national symbol.

**Figure 2:**
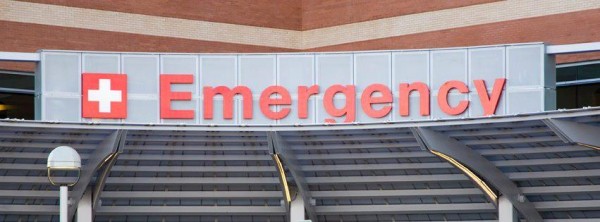
Entrance to the new emergency room at University of Colorado Hospital in Aurora, CO, mislabeled with the flag of Switzerland.

## The Swiss flag

**Figure 3:**
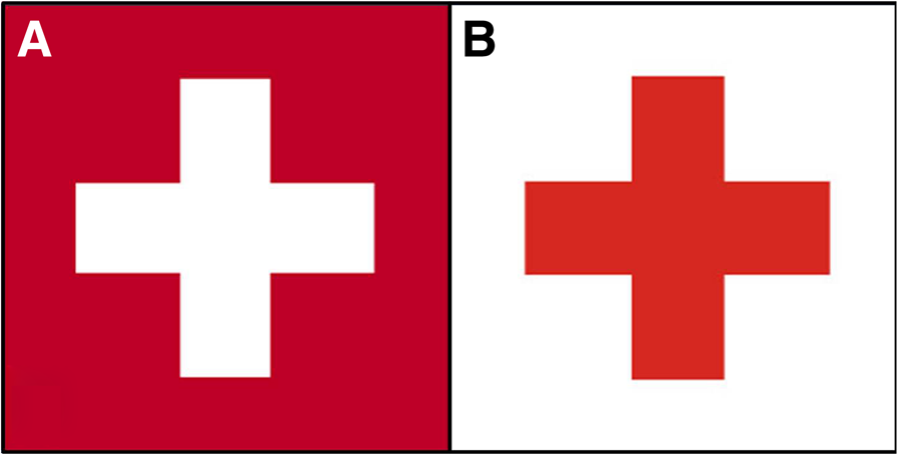
**Flag of Switzerland (A) and symbol of the Red Cross (B).** The design of the Red Cross originate from the First Geneva Convention in 1864. The symbol represents an inverted Swiss flag as a tribute to Henry Dunant, the Swiss founder of the *International Committee of the Red Cross*.

**Figure 4:**
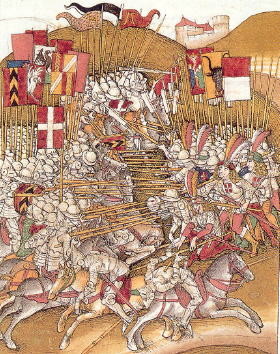
**First historic depiction of the Swiss flag at the Battle of Laupen, June 21, 1339.** Source: Illustrated Swiss chronicles by Diebold Schilling the Elder (1445–1485).

The flag of Switzerland consists of a symmetric white cross on red background (Figure 3A), and represents just one of two square-shaped national flags in the world (the other being the Vatican State) [2]. The design dates back to the 1300s, when Swiss Confederate troops started using a white cross on red background as their battlefield ensign (Figure 4). After being defeated by the French Army at the battle of Marignano in 1515, Switzerland pledged eternal neutrality [3]. Secondary to the lack of being further involved in military conflicts, the Swiss field sign fell out of use for more than three centuries. Occasional Swiss mercenaries were the exception who continued wearing the white cross insignia on battlefields during the 1600s and 1700s. When Switzerland officially became a sovereign federal state in 1848 [4], the national flag was introduced in present form (Figure 3A).

### **The Red Cross**

**Figure 5:**
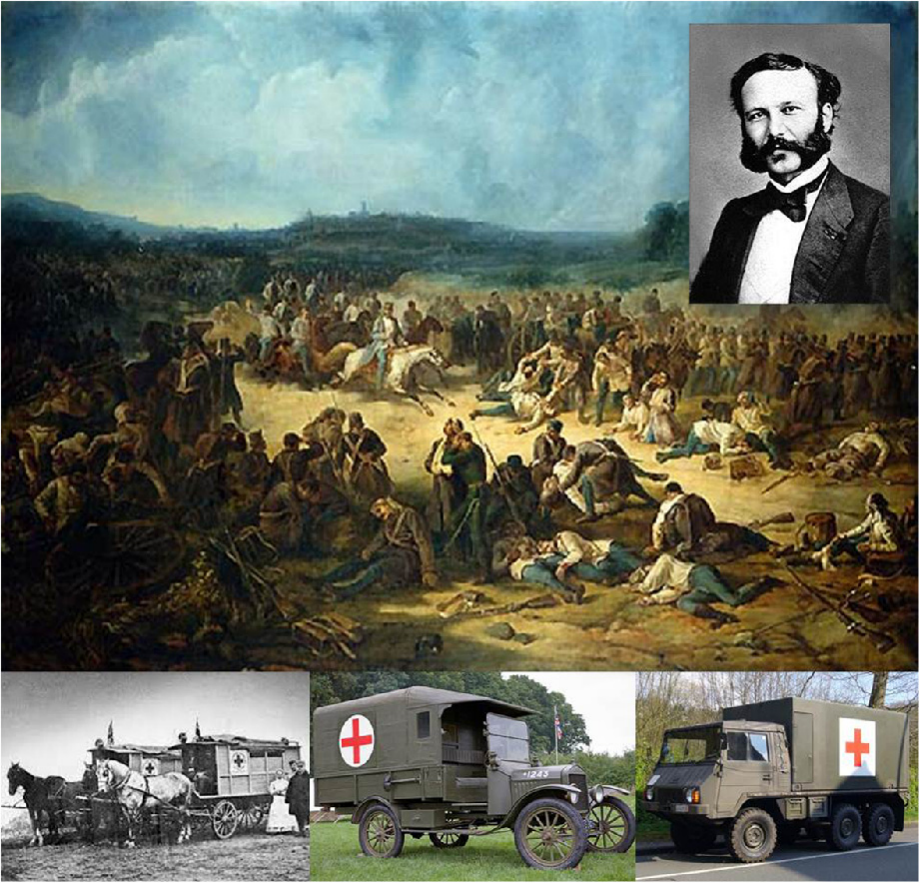
**Casualties from the Battle of Solferino (June 24, 1859) and evolution of military Red Cross ambulances.** The insert shows a contemporary picture of the Red Cross founder Jean-Henri Dunant (around 1860). The lower panels depict the development of Red Cross ambulances from a 19^th^ century horse carriage (left), to a world war I British Army Ford T model in 1916 (center), and a contemporary Swiss Army all-terrain Pinzgauer (right). *Source of the painting: “Battaglia di Solferino”, unknown painter, Museo Nazionale del Risorgimento Italiano, Torino, Italy.*

During the summer of 1859, the Swiss businessman Jean-Henri Dunant traveled to Northern Italy on a business trip. He accidentally witnessed the aftermath of the Battle of Solferino, a small town south to the Lake of Garda, in the evening of June 24, 1859 (Figure 5). More than 300,000 troops fought this monumental battle in a single day, which resulted in the defeat of the Austrian Army by an alliance of French and Sardinian troops. While touring the battlefield, Dunant observed more than 40,000 dead and wounded soldiers. He wrote a book entitled “*Un Souvenir de Solférino”* which described the aftermath of the battle and the suffering of the wounded soldiers deprived of medical care [5]. This work, in conjunction with Dunant’s tireless advocacy, inspired the creation of the *International Committee of the Red Cross* (ICRC) in 1863. The official Red Cross emblem was designed as the inverse of the Swiss flag [6], in honor of Dunant’s Swiss citizenship (Figure 3B). The founding of the ICRC laid the foundation to ameliorate the condition of wounded soldiers, and to guarantee the protection of neutral medics, ambulances, and field hospitals during armed conflicts. Jean-Henri Dunant was later awarded the first Peace Nobel Prize for this achievement in 1901. The current year marks the 150^th^ anniversary of founding of the ICRC, and the Red Cross has evolved to an internationally respected and trusted humanitarian symbol [7].

### Where does the confusion originate?

**Figure 6:**
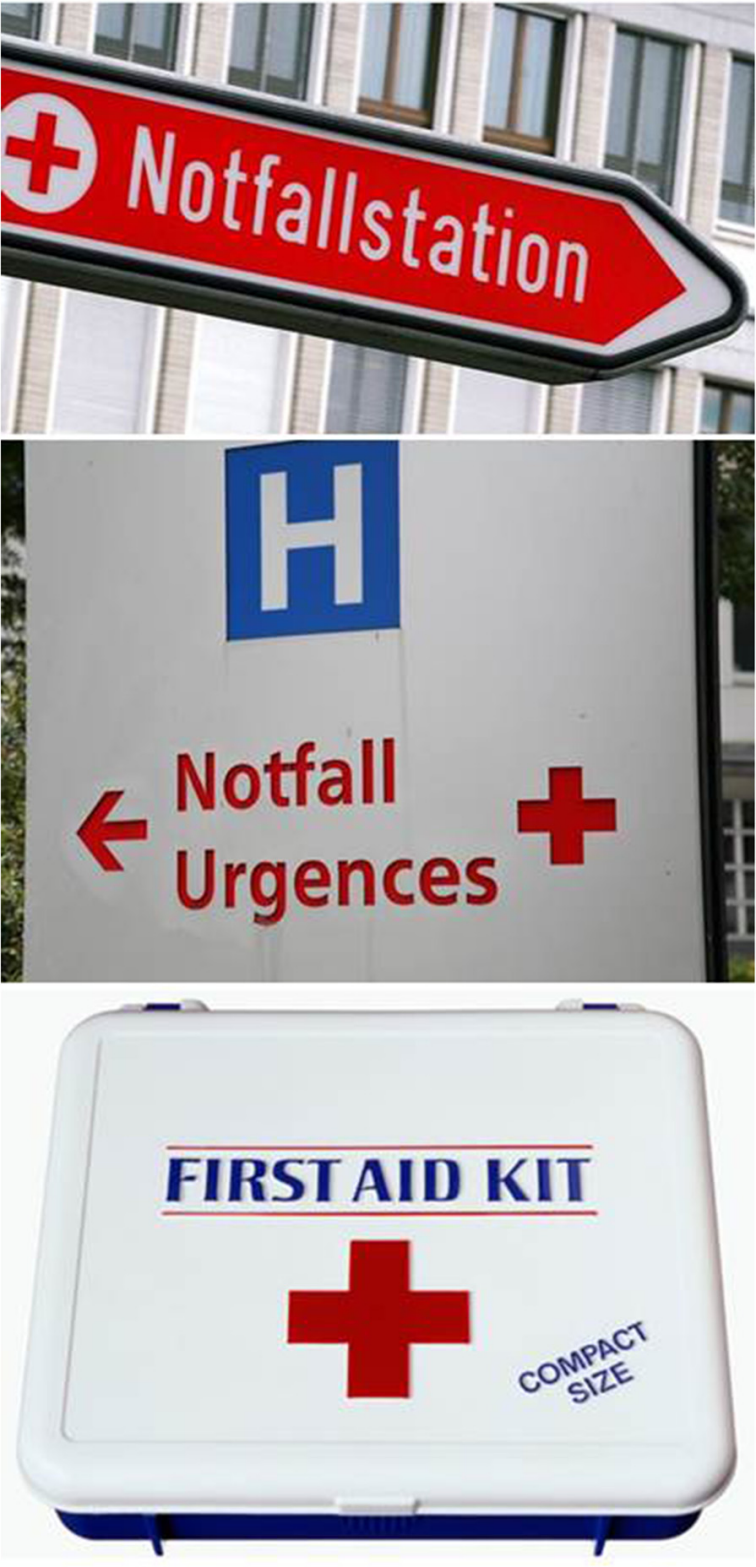
Examples of current generic use of the Red Cross emblem.

In light of the unequivocal definition of the two emblems for the Swiss flag and the Red Cross, respectively, I am puzzled as to the origin of the current widespread confusion between the two distinct symbols. Clearly, the Red Cross represents the neutral symbol of protection in wartime, reflecting Henri Dunant’s spirit of Solferino (Figure 5). In this regard, the main purpose of the Red Cross is to protect wounded victims of war and their designated caregivers. The use of the Red Cross was furthermore globally extrapolated to an attribute for a variety of healthcare-related entities, including hospitals, emergency rooms, first aid stations, first aid providers, and first aid kits (Figure 6). In contrast, the Swiss flag represents the national identification and sovereignty of Switzerland (in analogy to the *Stars&Stripes* flag for the United States).

From an international copyright law perspective, emblems of pure geometrical design, such as the Swiss flag or Red Cross, are not legally protected [8,9]. Only works defined as “creative art“ can be copyrighted, and an artwork must be sufficiently original to be eligible for copyright [8,9]. Common geometric forms are precluded from this definition. The appropriate use of national emblems (and its restrictions) is defined by official governmental orders and national laws. The same applies to the official authorization for the use of the symbol of the Red Cross, which is restricted by ICRC regulations, and by national Red Cross organizations and their respective local chapters and branches. Clearly, in light of its underlying humanitarian significance, the Red Cross emblem must be protected within specified limits from unauthorized use or misuse, whether by deliberate intention or inadvertent occurrence. Interestingly, a lawsuit over the use of the Red Cross symbol achieved recent international recognition, when the global pharmaceutical and medical device company *Johnson&Johnson* sued the American Red Cross over its use of the Red Cross trademark on commercial products [10,11]. This claim, which was settled after a longstanding dispute in 2008, supports the notion that legal copyright protection is unlikely affecting the appropriate humanitarian use of the Red Cross symbol, either in- or outside of the United States.

In summary, the initial question on the underlying root cause of the widespread confusion is best addressed by a classic quote from Sir Arthur Conan Doyle’s Sherlock Holmes [12]: *“When you have eliminated the impossible, whatever remains, however improbable, must be the truth.”* After eliminating legal ramifications as a potential deterrent from appropriate use of the Red Cross symbol, the remaining explanation is simple unawareness about the formal distinction from the Swiss national flag. Until formal clarification, we will continue to encounter emergency departments masked as Swiss embassy satellite offices (Figure 2), and ski patrollers disguised as Swiss citizens in the Rocky Mountains (Figure 1).

For feedback and further insight on the controversial topic discussed in this editorial, we encourage our readers to post a free comment through the article‘s weblink: http://www.pssjournal.com/content/7/1/13*> Tools > Post a comment.*

## Competing interest

The author is a Swiss citizen, and declares no other conflict of interest.
